# Pilot Screening of Cell-Free mtDNA in NIPT: Quality Control, Variant Calling, and Haplogroup Determination

**DOI:** 10.3390/genes12050743

**Published:** 2021-05-14

**Authors:** Alisa Morshneva, Polina Kozyulina, Elena Vashukova, Olga Tarasenko, Natalia Dvoynova, Anastasia Chentsova, Olga Talantova, Alexander Koroteev, Dmitrii Ivanov, Elena Serebryakova, Tatyana Ivashchenko, Aitalina Sukhomyasova, Nadezhda Maksimova, Olesya Bespalova, Igor Kogan, Vladislav Baranov, Andrey Glotov

**Affiliations:** 1 D.O. Ott Research Institute for Obstetrics, Gynaecology and Reproductology, Mendeleevskaya Line 3, 199034 St. Petersburg, Russia; polykoz@gmail.com (P.K.); vi_lena@list.ru (E.V.); olgatar777@mail.ru (O.T.); olga_talantova@mail.ru (O.T.); el.a.serebryakova@mail.ru (E.S.); tivashchenko2011@mail.ru (T.I.); shiggerra@mail.ru (O.B.); ikogan@mail.ru (I.K.); vsbar40@mail.ru (V.B.); anglotov@mail.ru (A.G.); 2 Ltd NIPT, Bolshoi V.O. 90, Building 2 lit. 3, 199106 St. Petersburg, Russia; 1.cosulya@gmail.com (N.D.); nastya.chentsova@gmail.com (A.C.); 3 St. Petersburg State Pediatric Medical University, 2 Litovskaya Street, 194100 St. Petersburg, Russia; alexkoroteev@mail.ru (A.K.); spb@gpma.ru (D.I.); 4 Center for Medical Genetics, Tobolskaya ul. 5, 194044 St. Petersburg, Russia; 5 Molecular Medicine and Human Genetics, Research Laboratory, Medical Institute, M.K. Ammosov North-Eastern Federal University, 677007 Yakutsk, Russia; aitalinas@yandex.ru; 6 Republican Hospital No. 1, National Medical Centre, Ministry of Public Health of the Sakha Republic, 677008 Yakutsk, Russia; nogan@yandex.ru

**Keywords:** NIPT, foetal fraction, transportation, cfDNA, mtDNA, mitochondrial diseases, mitochondrial variants, SNPs, ClinVar, breast cancer, mtDNA haplogroups, population studies

## Abstract

Clinical tests based on whole-genome sequencing are generally focused on a single task approach, testing one or several parameters, although whole-genome sequencing (WGS) provides us with large data sets that can be used for many supportive analyses. In spite of low genome coverage, data of WGS-based non-invasive prenatal testing (NIPT) contain fully sequenced mitochondrial DNA (mtDNA). This mtDNA can be used for variant calling, ancestry analysis, population studies and other approaches that extend NIPT functionality. In this study, we analyse mtDNA pool from 645 cell-free DNA (cfDNA) samples of pregnant women from different regions of Russia, explore the effects of transportation and storing conditions on mtDNA content, analyse effects, frequency and location of mitochondrial variants called from samples and perform haplogroup analysis, revealing the most common mitochondrial superclades. We have shown that, despite the relatively low sequencing depth of unamplified mtDNA from cfDNA samples, the mtDNA analysis in these samples is still an informative instrument suitable for research and screening purposes.

## 1. Introduction

Discovery of the foetal DNA in maternal plasma made in 1997 by Denis Lo has marked the beginning of a new era in prenatal testing [1]. Since the introduction of non-invasive prenatal testing (NIPT) into clinical practice in 2011, initially as cell-free DNA (cfDNA)-based testing for foetal Down syndrome detection, the functionality of the method is constantly expanding [2]. Current diagnostic possibilities of NIPT cover a wide variety of foetal aberrations, such as common and rare trisomies, sex chromosome abnormalities (SCAs), deletions and microdeletions, duplications, copy number variants (CNVs), monogenic disorders, etc., and there is still place for improvement [2,3,4,5].

NIPT based on whole-genome sequencing (WGS) has especially high diagnostic and research potential since a large amount of sequenced cfDNA data enable many diagnostic instruments and can be further used for conducting various supporting studies for both the mother and foetus [6,7]. Low genome coverage remains the principal limitation that hampers the development of NIPT extensions. However, cfDNA fraction was reported to contain the full mitochondrial genome [8], compared to fairly low nuclear genomic DNA coverage. This can be explained by the higher copy number and relatively small size of mitochondrial DNA (16,569 bp) in comparison to genomic DNA (3 Gbp) [9]. Mitochondrial genes can undergo natural transfer to nuclear DNA so the nuclear copies of mtDNA (NUMTs) are formed [10]. NUMTs can be a source of contamination in mtDNA analyses [11] and compromise the results. Nevertheless, as mentioned, the low nuclear genome coverage in NIPT samples reduces the impact of such effects.

The fact that mitochondrial DNA (mtDNA) in NIPT samples is fully covered opens the door to variant calling and mtDNA analysis across many samples. The pool of mtDNA in cfDNA fraction is heterogeneous and contains fragmented mtDNA from apoptotic cells of different types and, in case of pregnancy, foetal mtDNA [12]. Due to its prokaryotic origin, mtDNA has high inflammatory potential since it is similar to bacterial DNA and triggers an anti-pathogen response [13]. MtDNA, along with ATP and cytochrome-c, belongs to damage-associated molecular pattern molecules (DAMPs) working as endogenous danger signals and releasing from damaged mitochondria of dying cells or injured tissues [14,15]. The release of mitochondrial DAMPs into the circulation is a marker of systemic inflammatory responses and a direct link between elevated cell-free mtDNA (cf-mtDNA) level and chronic inflammation in patients with type 2 diabetes [16]. Alterations in the cf-mtDNA level were also reported for many chronic diseases and pathologic states, such as sepsis, injuries, diabetes, coronary heart diseases, Parkinson’s disease and Alzheimer’s disease [17,18,19,20,21].

Due to continuous apoptosis of placental trophoblasts, pregnancy can be viewed as a pseudoinflammatory state [22]. As a result, mtDNA is detectable in maternal plasma in normal pregnancy and increased in cases of many pregnancy complications [23,24,25,26]. This increases the interest of clinicians in cf-mtDNA fraction due to its potential application in diagnostics of pregnancy pathologies such as preterm birth that is known to be tightly connected with inflammation [27].

Furthermore, a lot of mitochondrial variants are proved or presumed to be associated with various diseases. These variants might affect energy metabolism, production of reactive oxygen species (ROS), ATP synthesis, calcium signalling and apoptosis but the exact mechanisms behind the observed associations are still to be uncovered [28,29,30,31]. Mitochondrial mutations underlie such pathologies as Leber hereditary optic neuropathy (LHON), Leigh syndrome (LS), neurogenic muscle weakness, ataxia and retinitis pigmentosa (NARP) and many others [32,33,34]. Since cells contain many copies of mitochondria, one cell can contain both mutated and normal mtDNA—this heterogenic state is known as heteroplasmy [35]. Heteroplasmic state is typical for most of the mitochondrial variants, including pathogenic ones and disease severity is often associated with the amount of mutated mtDNA in cells [36]. 

Apart from its diagnostic capabilities, mtDNA is of great interest for population and phylogenetic studies since it is not as conservative as nuclear DNA and deteriorates much faster with an average mutation rate of ~2.7 × 10^−5^ per base per 20-year generation [37] against ~2.5 × 10^−8^ per base per generation mutation rate of genomic DNA [38]. Notably, the speed of mutation varies between different regions of mtDNA, reaching the maximum in the non-coding control region, which accumulates the majority of all mitochondrial SNPs [39]. Non-coding D-loop region (16,024−576 bp) is a hot spot for mtDNA alterations, containing two hypervariable regions (HV1 at 16,024–16,383 bp and HV2 at 57–372 bp) [40]. High mutability of mtDNA and its maternal inheritance make mtDNA exceptionally useful as a molecular clock.

MtDNA along with Y chromosome is the common trackers of human ancestry [41,42], both having the uniparental type of inheritance and representing maternal and paternal lineage respectively. MtDNA carries a particular combination of variants inherited from a common ancestor, defining its haplotype [43]. Human mitochondrial phylogenetic trees have these mitochondrial haplotypes as the leaves, which can be assigned to mitochondrial haplogroups [44]. Mitochondrial haplogroups are widely used to track the population origins and genetic structure. This can be especially interesting for exploring the structure of mixed populations. 

There are works demonstrating the possibility of using low-coverage WGS sequencing NIPT data for population studies, but they are mostly focused on genomic DNA [45,46].

In this study, we work with samples from different regions of Russia, including for the most part Northwestern, Central, Volga-Ural regions and Yakutia, which is populated by the indigenous ethnic groups of the Mongoloid (Asian) race (54.1%): Yakuts and minor groups of Evenks, Evens, Dolgans, Yukagirs, Chukchi and non-indigenous ethnic groups (45.9%), including the Caucasians Russians and Ukrainians. Yakuts is one of the Turkic ethnic groups that emerged as a mixture of Turkic people and indigenous people [47,48]. Phylogenetic analysis has revealed that indigenous ethnic groups belong to the E Asian clade or the Beringian–American clade, including native Americans [49,50]. Thus the heterogeneity of ethnic groups in our dataset can be of interest for both populational and clinical studies.

Analysis of mtDNA underlies many medical and genetic tests. Apart from maternal ancestry testing, based on mtDNA haplogroup determination [51], there are genetic diagnostic tests for mitochondrial diseases, mainly running on mtDNA whole genome sequence [52] or a panel of common mtDNA point mutations associated with various mitochondrial disorders [52,53].

In this study, we explore the utility of using NIPT data for mtDNA studies. Given the increasing popularity of NIPT, the possibility of the analysis of mtDNA from cfDNA data is quite promising.

## 2. Materials and Methods

### 2.1. Data

In this study, we processed 645 samples from patients with both normal and pathologic pregnancies subjected to NIPT analysis in The Research Institute of Obstetrics, Gynecology and Reproductology named after D.O. Ott. Samples were transported from different regions of Russia: 397 samples from Northwestern district (mostly Saint-Petersburg), 120 from Central district, 63 from Volga-Ural district, 49 from Yakutia and 10 from North Caucasian districts. Southern and Far Eastern districts are presented by 4 and 2 samples respectively. All women signed informed consent for studies and processing of personal data, including medical history data. The study was performed in accordance with the Declaration of Helsinki.

### 2.2. Plasma DNA Isolation

Blood samples were collected either in tubes with 0.5 M EDTA solution (pH = 8.0) (Greiner Bio One, Kremsmünster, Austria) or Cell-Free DNA Streck^™^ BCT^®^ blood collection tubes (cfDNA BCT, La Vista, NE, USA). EDTA tubes were centrifuged 30 min after the blood sampling at 2000 *g* for 10 min at 4 °C to get plasma and then centrifuged the plasma at 16,000 *g* for 10 min at 4 °C. Streck tubes were centrifuged on arrival from regions at 1600 *g* for 10 min at room temperature to get plasma and then the plasma was centrifuged at 16,000 *g* for 10 min at room temperature. For plasma from Streck tubes, we used a Proteinase K treatment step (≥30 mAU/mL digest) at 60 °C in the presence of SDS for 1 h when extracting cf-DNA. For DNA extraction we used ‘MagMAX Cell-Free DNA Isolation Kit’ (Thermo Fisher Scientific Inc., Waltham, MA, USA) following the manufacturer’s instructions. 

### 2.3. Buffy Coat Analysis

In order to explore the differences between mtDNA pools of buffy coat and cfDNA from blood plasma, we have sequenced DNA, extracted from several buffy coats stored either in EDTA (n = 4) or Streck Cell-Free DNA BCT blood collection tubes (n = 4). Each one of buffy coat DNA samples has been paired with the corresponding cfDNA sample. DNA samples from the buffy coats were isolated by salt-out extraction with some modifications [54]. Briefly, buffy coats were resuspended in nuclei lysis buffer (10 mM Tris-HCl, 400 mM NaCl and 2 mM Na2EDTA, pH 8.2). The cell lysates were digested 5 h at 55 °C with 0.02 mL of 10% SDS, 0.4 mL of a proteinase K solution (10 mM Tris-HCl, 0.15 M NaCl and 1 mM Na2EDTA, pH 8.0) with 21 mg proteinase K. After digestion was complete, 1 mL of 5 M NaCl was added and shaken vigorously for 15 s. Then one volume of chloroform was added and shaken for 10 min followed by centrifugation at 10,000 rpm for 10 min. The upper aqueous phase was transferred to a fresh tube. Then one volume of chloroform was added again and shaken for 5 min followed by centrifugation at 10,000 rpm for 5 min. The upper aqueous phase was transferred to a fresh tube. Two volumes of room temperature absolute ethanol were added and the tubes inverted several times until the DNA precipitated. The DNA pellet was washed with 70% ethanol twice, and was collected at the bottom of the tube by centrifugation at 5000 rpm for 5 min and dried at room temperature. The DNA was allowed to dissolve in 0.2 mL TE. Before preparing the libraries, DNA fragmentation was performed using the Diagenode Bioruptor UCD-200 according to the manufacturer’s recommendations.

### 2.4. Library Preparation & Sequencing

The library preparation step involved three steps: blunt-end ligation, barcode ligation and amplification. We used the ‘Ion Plus Fragment Library Kit’ (Thermo Fisher Scientific Inc., USA) protocol with some proprietary modifications (patent G01N 33/48, C40B 50/00, G06F 19/20, C12Q1/68). DNA concentrations were measured with ‘Qubit 2.0’ fluorimeter (Invitrogen, Carlsbad, CA, USA), using ‘Qubit dsDNA HS Assay Kit’ (Thermo Fisher Scientific Inc., USA). The quantity of libraries required for sequencing was determined according to the manufacturer’s protocol (Thermo Fisher Scientific Inc., USA). Quality of samples has been tested with capillary electrophoresis ‘TapeStation Instrument’ (Agilent technologies, Santa Clara, CA, USA), using ’High Sensitivity D1K ScreenTape’ and ‘High Sensitivity D1K Reagents’ (Agilent technologies, USA). ‘Ion Chef System’ (Thermo Fisher Scientific Inc., USA) has been used for sample loading with ‘Ion 540 Kit-Chef’ (Thermo Fisher Scientific Inc., USA) following the manufacturer’s instructions. We used microchips ‘Ion 540 Chip’ (Thermo Fisher Scientific Inc., USA) following the manufacturer’s instructions. Sequencing has been performed with ‘Ion Torrent S5’ (Thermo Fisher Scientific Inc., USA) with the average coverage of 3–4 million reads per sample. The preliminary processing of sequencing results was performed using Torrent Suite software v5.12.2 (ThermoFisher Scientific Inc., USA). The bam files were filtered by sequencing quality (trimming quality cutoff 15) and barcodes and adapters trimmed off. 

### 2.5. Variant Calling and ClinVar Annotation

For each sample, we extracted mtDNA reads from Ion Torrent S5 generated WGS bam files using Samtools [55]. Mitochondrial variants were called using MuTect2 from GATK4 [56,57] in the mitochondria mode. Variants recovered in VCF files were filtered by sequencing depth (<5) for further analysis to exclude variants with low depth; variants located within homopolymers (of length 4 and more) were filtered out with VCFPolyX [58]. For variant annotation, we used the ClinVar database of 16 March 2020 [59] and VariantAnnotator from GATK4 with default parameters and hg19 as a reference genome [60].

### 2.6. Haplogroup Assignment

Haplogroups of mtDNA were assigned with HaploGrep2 [61] based on the sets of variants found in individual samples, samples with the lowest haplogroup confidence score (0.5) were discarded. 

## 3. Results

### 3.1. MtDNA Pool Is Better Presented in cfDNA Samples from Streck Collection Tubes

**Figure 1:**
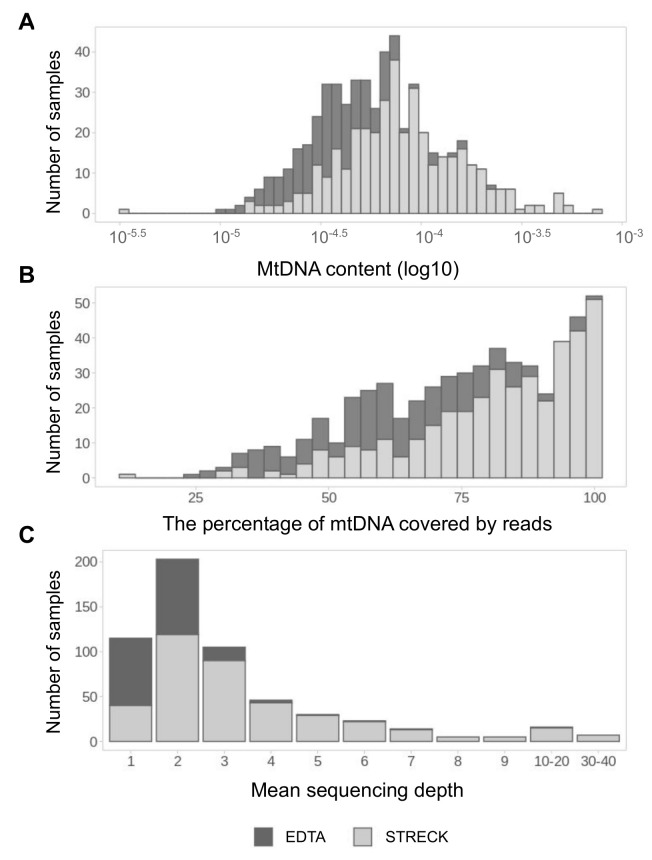
The quantitative analysis of the mtDNA pool in samples. MtDNA content (**A**), mtDNA coverage (**B**) and sequencing depth (**C**) distribution in samples collected to EDTA (dark-grey) and Streck (light-grey) sample tubes.

At the outset, we conducted a quantitative analysis of mtDNA fraction of 645 cfDNA samples of pregnant women. MtDNA content was measured for each sample as the ratio of mitochondrial reads to the total number of reads in the sample. The number of mitochondrial reads varies from 16 to 5536, with a median of 276 mtDNA reads per sample. The mtDNA content of different samples varies by two-three orders of magnitude and generally is higher in samples collected to Streck tubes than in samples collected to EDTA tubes (Figure 1A). The same trend can be observed for the mtDNA coverage: the vast majority of samples with more than 75% mtDNA coverage belongs to the Streck group (Figure 1B). In total, 57% of all samples have mtDNA coverage exceeding 75%. The mean sequencing depth is also demonstrating the same trend. The mean sequencing depth of different samples varies from 1 to 40 (Figure 1C). In general, Streck samples have a better quality of sequenced mtDNA material.

### 3.2. Variant Calling Reveals a Number of Non-Ancestral Mitochondrial Variants Frequent within the Russian Population 

All 645 mtDNA samples have been processed for mitochondrial variant calling. Variant calling was performed using MuTect2 from GATK4 in the mitochondria mode, which basically works like a somatic variant caller. From these samples we called 32962 variants. To reduce the impact of sequencing errors, we performed a two-step filtration (Table 1). First of all, variants with sequencing depth of less than 5 were filtered out. The depth threshold for mitochondrial variants is usually lower than the one for genomic variants since mitochondrial variants can be presented in a small fraction of reads due to heteroplasmy and high copy number. Next, we filtered out the variants found within homopolymers since the semiconductor sequencing platform is prone to errors in homopolymer regions. We excluded variants within polynucleotide repeats of the length 4 and more, which were mostly indels. Due to strict filtering conditions, we missed a certain percentage of the true variants, however, we were still able to successfully detect a good part of them. 

**Figure 2:**
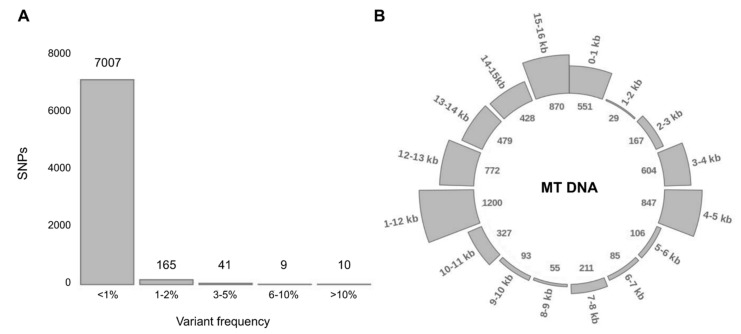
Variant distribution: frequency and location in the mitochondrial genome. (**A**) Distribution of distinct variants according to their population frequency (by the percentage of samples carrying the particular variant). (**B**) Distribution of variants throughout the mitochondrial genome. Figures outside the circle mark position in the mitochondrial genome (16 kb mtDNA has been divided into 16 sections per 1 kb), figures in the inner circle represent the number of variants in every section.

Remaining pool of variants (14681) is represented by 7493 distinct variants. Most of them (96.2%) have a frequency of less than 1% (Figure 2A) and only 10 variants exceed 10% frequency. Among the detected variants, 6 have population frequency exceeding 15%: variants m.9769delT (23.3%), m.15326A>G (22.3%), m.750A>G (20.9%), m.2706A>G (18.8%), m.12272delA (15.7%), m.263A>G (15.0%) are the most frequent in all explored geographic regions. A majority of them belong to ancestral variants, common across all lineages and especially frequent in lineages of superclade M, including lineages C and D [62]. 

Aside from ancestral variants, reasonably frequent in all populations, we selected the top-5 of non-ancestral point (Table 2) and indel (Table 3) variants frequent in our data. For indels, we used stricter homopolymer filtering (≥3) since most of the frequent indels are located within homopolymers of length 3 and more and can stem from sequencing errors (Table 1). 

Since most of these variants are not presented in databases, we also highlight the top-5 of ClinVar SNPs (Table 4).

As expected, the distribution of detected variants throughout the mitochondrial genome is not uniform. There are so-called ‘hot spots’ in 3–5, 11–13 and 15–16 kb (Figure 2B), carrying 66.9% of all variants and ‘cold spots’ in 1–2, 5–7 and 8–10 kb, carrying only 5% of variants. Variants are accumulating in upstream and downstream gene regions (intergenic variants).

Due to strict filtering conditions, a certain percentage of the true variants are inevitably missing, especially in homopolymeric regions. Nevertheless, the proportions of variants in individual sections of the mitochondrial genome are not affected by filtering (Figure S1).

### 3.3. The Buffy Coat and cfDNA Contain Different Pools of mtDNA

In order to assess the contribution of degraded blood cells to the overall mtDNA pool, we compared mtDNA pools between cfDNA samples and corresponding buffy coat samples. Sequenced data obtained from buffy coat DNA and cfDNA samples proceeded through the same steps: extraction of mtDNA reads, estimation of mtDNA rate, variant calling and filtering of homopolymer variants.

**Figure 3:**
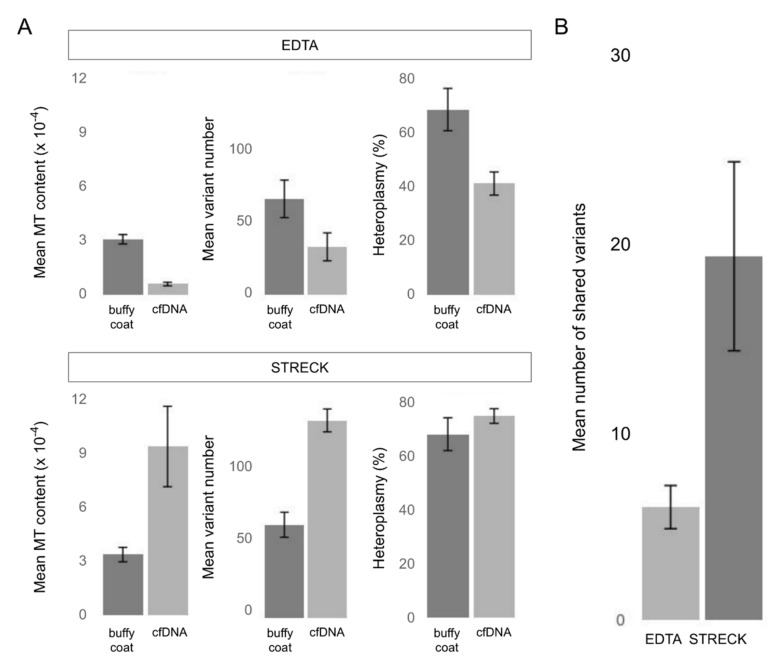
The comparison between mtDNA pools of buffy coat and cfDNA from blood plasma in samples with different transportation conditions (transported in EDTA (blood plasma) or Streck (whole blood) sample tubes). The bars represent the mean values for the buffy coat—cfDNA pairs (n = 4); error bars indicate the standard error of the mean (SEM). (**A**) Average mtDNA content (the ratio of mtDNA reads to nuclear DNA reads averaged over all samples), the average number of called mitochondrial variants (homopolymer variants were filtered out) and the average percentage of heteroplasmy in buffy coat and cfDNA samples. (**B**) The average number of the mitochondrial variants shared by mtDNA pools of buffy coat and cfDNA (homopolymer variants were filtered out).

The comparison of such parameters as mtDNA content, number of called variants and percentage of heteroplasmy within pairs has revealed different trends for samples stored in EDTA and Streck sample tubes. Within these groups of sample pairs (the EDTA group and the Streck group), pairs demonstrated similar parameters. Therefore, we averaged the values within each group and compared the common characteristics of mtDNA pools from buffy coat and cfDNA separately for EDTA and Streck (Figure 3A).

Our results show that cfDNA extracted from samples transported as blood plasma (EDTA) contains significantly less mtDNA than DNA from buffy coat and therefore have a smaller set of mitochondrial variants, while cfDNA extracted from samples transported as whole blood (Streck) are enriched with mtDNA and consequently carry more variants. The heteroplasmy level has also demonstrated the same trend. However, despite lower mtDNA content, the mtDNA pool in EDTA samples is still enough for the analysis.

To evaluate the similarity of mtDNA pools from buffy coat and cfDNA, we estimated the number of variants they share. According to our results, sets of mitochondrial variants called from buffy coat and cfDNA of one individual are quite different and have a little overlapping, suggesting that buffy coat and cfDNA contain different pools of mtDNA which can be attributed to the mixed origin of cf-mtDNA. MtDNA from the buffy coat samples stems from the blood cells, representing the limited set of cell populations. Notably, the Streck samples of buffy coat DNA and cfDNA share more variants, indicating that degrading blood cells make a significant contribution to the pool of cf-mtDNA (Figure 3B). On average, Streck samples share three times more variants. Thus the difference between two types of sample tubes can be essential to downstream NIPT analysis and needs further investigation.

### 3.4. Long Transportation Results in an Increase of mtDNA Content which Leads to the Lower Fetal Fraction in Samples

**Figure 4:**
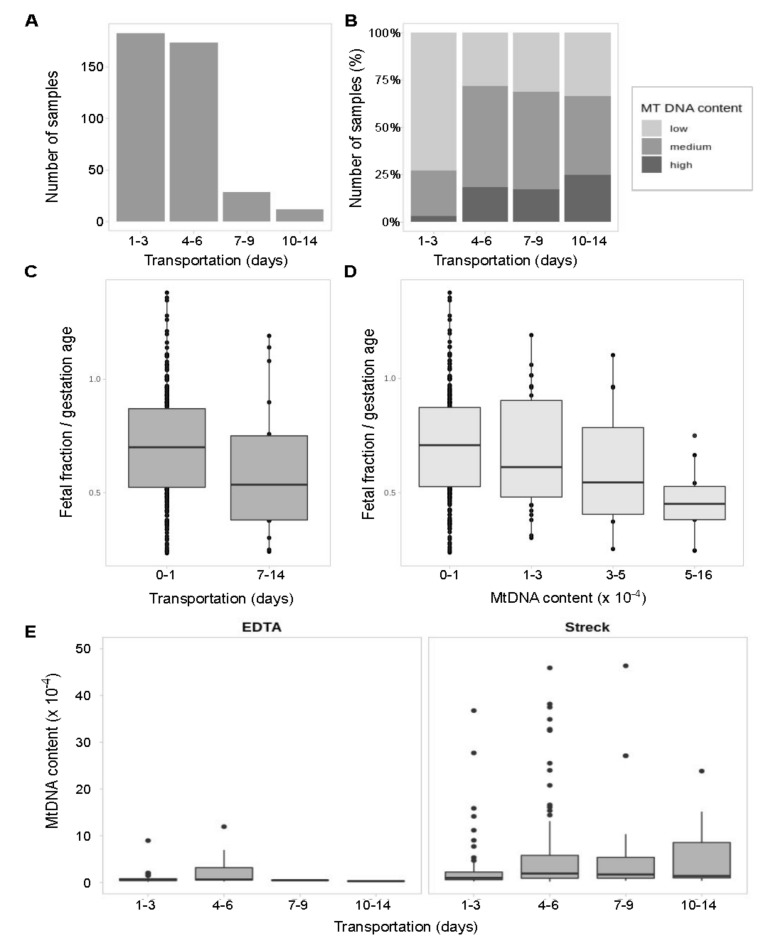
MtDNA content depending on storage conditions and transportation. Statistical significance has been measured with the Kruskal–Wallis test. (**A**) Distribution of samples according to time of transportation (days). (**B**) Distribution of samples with low, medium and high mtDNA content within each time period (percentage). (**C**) Foetal fraction scaled to gestation age in samples shipped under or over 7 days. No statistically significant difference found (*p* = 0.09). (**D**) Foetal fraction scaled to gestation age in samples grouped by mtDNA rate. The difference is statistically significant (*p* = 0.007). (**E**) Temporal dynamic of mtDNA content in cfDNA fraction extracted from samples stored in EDTA (blood plasma) or Streck (whole blood) sample tubes. The difference is significant for Streck samples (*p* = 0.0014).

Our study includes samples from remote regions, so to check the possibility of blood cell DNA leakage into a cell-free fraction we explored the effect of long transportation on mtDNA content and hence on the number of called variants. For different samples, time of transportation varies from 1 to 14 days (Figure 4A). Content of mtDNA in samples increases over time (Figure 4B).

Among the samples shipped within 72 h, samples with low mtDNA content prevail and samples with high amounts of mtDNA are almost never met. After that 72-h threshold, the fraction of samples enriched with mtDNA rises. Notably, this mtDNA enrichment is observed only in Streck Cell-Free DNA BCT blood collection tubes storing whole blood. As expected, EDTA tubes storing blood plasma show equal mtDNA content over time (Figure 4C) supporting the hypothesis that DNA from degraded blood cells accumulates in Streck Cell-Free DNA BCT blood collection tubes over time.

The previous studies [63,64] have explored the stability of DNA in EDTA and Streck collection tubes but only for the short periods of 72 h and less. We assumed that the longer the transportation time the more blood cells get degraded, which might negatively affect the foetal fraction level and thus potentially affect the quality of NIPT results. To check this out, we evaluated the difference between the foetal fraction size in samples shipped under and over 7 days. Indeed, there is a downward trend in the foetal fraction size after 7 days of transportation (Figure 4D) and the statistically significant decrease of foetal fraction level in samples with high mtDNA rate (Figure 4E).

### 3.5. Analysis of Population Frequencies Reveals Differences in Top-Frequent Variants between Regions of Russia

**Figure 5:**
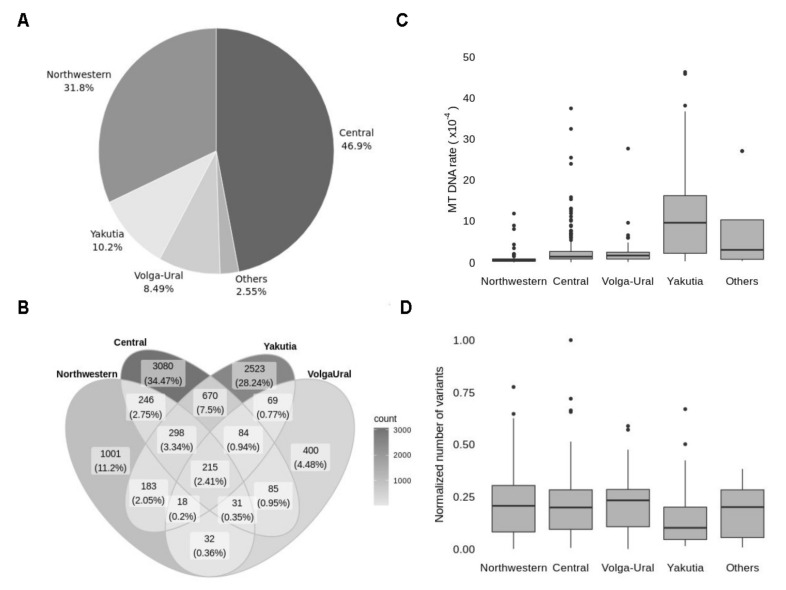
Geographic origin of explored samples. (**A**) Distribution of samples according to geographic region (Others include Southern, Far Eastern and North Caucasian regions, presented with a minor fraction of samples). (**B**) A Venn diagram representing the intersection between sets of variants in four regions: Northwestern, Central, Volga-Ural and Yakutia. Figures represent the number of distinct variants in every section and the percentage of the total number of variants. (**C**) Distribution of samples in every region according to mtDNA content. (**D**) Distribution of samples in every region by the number of variants, normalised to mtDNA content.

To explore the cross-regional differences, we split samples into groups according to their origin. The vast majority of samples were taken from Central (46.9%) and Northwestern (31.8%) regions, 10.2% were transferred from Yakutia, 8.49% from Volga-Ural region, the rest 2.55% of samples (‘Others’ category) were transferred from Southern, North Caucasian and Far Eastern regions (Figure 5A).

The variant intersection between Central, Northwestern, Yakutia and Volga-Ural regions has revealed only 215 common variants, which is only 2.4% of all detected variants (Figure 5B). The most frequent variants in each region belong to this 2.4% of shared variants and mostly presented by frequent ancestral variants, so explored regions do not have unique mitochondrial markers. However, the frequency of some variants in Yakutia reaches over 70% while in other regions these variants do not exceed 10% frequency or are even absent (Table 5). Non-ancestral variants frequent in the Yakutian population are included in the top of the most frequent variants for lineage M in MITOMAP (which refers to Mongolian ancestry), confirming the validity of our results.

Since Northwestern and Central regions are widely presented in our data, the large sets of variants for these regions were quite expected. To the contrary, 40% of detected variants originate from Yakutia, which accounts for only 10% of the samples. Since we established that long transportation affects the number of detected variants due to increased mtDNA content, we explored mtDNA content in samples from different regions. Distribution of mtDNA content (Figure 5C) between regions is in line with the distribution of the variants. Increased mtDNA content is observed in the most remote regions, so despite the low number of samples, these regions are making a large proportion of variants. To ensure that there is no real difference in the number of variants between regions, we normalised the number of variants to mtDNA content for each sample (Figure 5D). 

### 3.6. MtDNA Haplogroup H along with C, D and U Are the Most Frequent among Explored Samples

Haplogroups of mtDNA for individual samples were identified with HaploGrep [61] based on the filtered sets of variants. Variants located within homopolymer regions were filtered out as described in Section 3.2.

**Figure 6:**
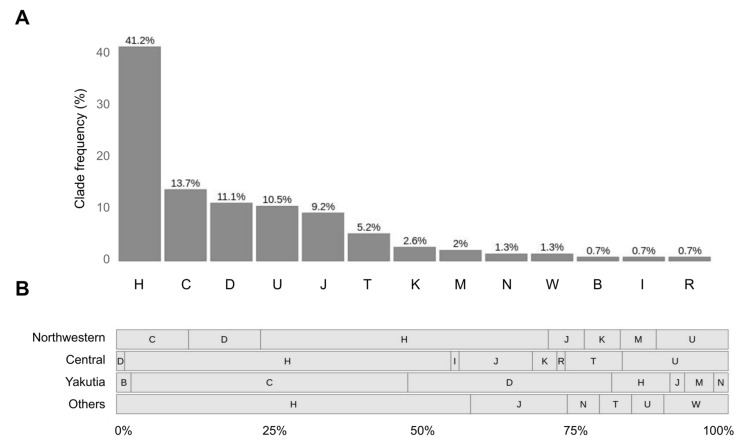
Haplogroup analysis with HaploGrep2. (**A**) Distribution of samples according to their superclade. (**B**) Distribution of samples from different regions according to their superclade. Others include Volga-Ural, Southern, Far Eastern and North Caucasian regions. The bars indicate the proportion of each superclade (percentage from the total number of samples in each region).

Analysis of haplogroup frequencies at the level of clades has revealed that haplogroups H (41.2%), C (13.7%), D (11.1%) and U (10.5%) are prevalent (Figure 6A). The rest of the haplogroups do not exceed 10% frequency—J (9.2%), T (5.2%), K (2.6%) and minor haplogroups M, N, W, B and I, presented by isolated findings and together make up 6% of all samples. To improve the reliability of the results in view of the low coverage data, samples with the lowest haplogroup confidence score (0.5) were discarded. These were mostly samples presented by less than 40 variants.

Next, we explored how this distribution changes from region to region (Figure 6B). We revealed that haplotypes of H superclade are top-frequent in all regions except for Yakutia, where most samples have haplogroups of superclades C (45%) and D (33%).

For some samples enriched with mtDNA we determined full haplogroup with high quality score: 19 samples with the quality score exceeding 85% and 4 variants around 90%-H2a1c (91%), J1c2 (90%), D4I2a1 (89%), H1c4b (89%).

### 3.7. The Vast Majority of Called Variants Are Reported to Be Benign According to ClinVar Database

Mitochondrial variants were then annotated using ClinVar database [59]. It must be noted that some diagnoses from ClinVar database are yet to be validated. In this respect, we cannot draw compelling conclusions on the actual pathogenicity of the variants found. For common variants, we verified ClinVar diagnoses from independent sources wherever possible.

**Figure 7:**
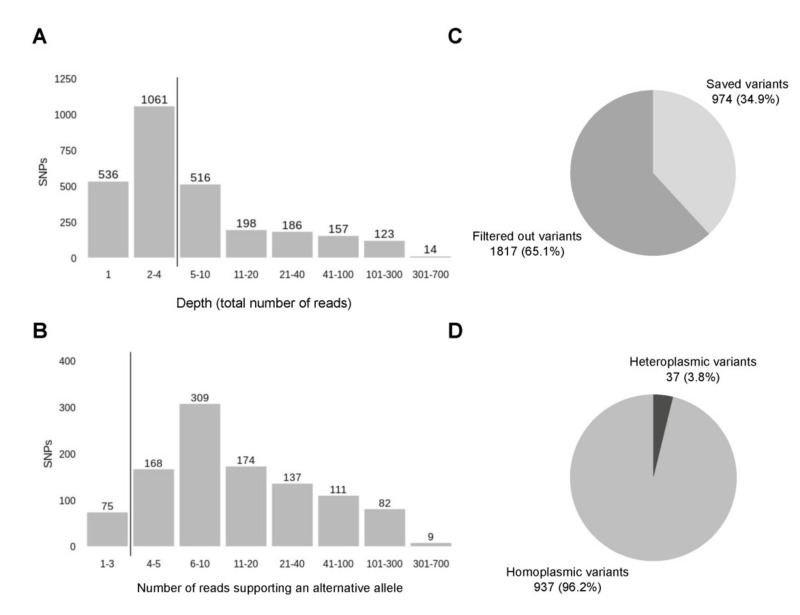
Filtration of the variants. (**A**) The first step of filtration or filtering out the variants with the lowest depths (less than 5), the vertical line sets the threshold. (**B**) The second step of filtration or filtering out the variant with the low number of reads carrying an alternative allele, the vertical line sets the threshold in 4 reads. (**C**) The distribution of variants after two steps of filtration. (**D**) The distribution of filtered variants by the number of reads supporting a reference allele—homoplasmic (all reads carry an alternative allele) and heteroplasmic (there are both reads with alternative and reference allele).

After ClinVar annotation, we have got 2791 variants. The depth of detected variants ranges from 1 to 647, where 4 is the median (Figure 7A). Variants with low depth (<5) and variants located within homopolymers were filtered out as described before (Section 3.2) that reduced the number of variants to 1065. Notably, the low-depth regions and homopolymers are not the only factors that need to be considered, because depth represents a total number of reads and consists of reads with reference and alternative allele. Since a low amount of reads carrying the variant of the alternative allele can stem from the sequencing errors outside of homopolymer regions, we included an additional step of filtration, filtering out the variants with less than 4 reads supporting an alternative allele (Figure 7B). During this two-step filtration, 1817 (65.1%) of all variants were discarded, all steps of the following analysis were performed for the remaining 974 (34.9%) variants (Figure 7C). Analysis of the alternative allele frequencies (percentage of reads supporting an alternative allele) has revealed that the majority of variants detected–96.2%–are represented by an alternative allele only and can be considered homoplasmic. In fact, the rate of homoplasmic variants is even higher if, by analogy with minimisation of possible sequencing errors by filtering out the variants with a low number of alternative reads, we accept that low representation of reads with a reference allele can also stem from sequencing errors. Since both of these two outcomes are equally likely, variants, where a reference allele is supported by a low number of reads (less than 4), can be also counted as homoplasmic. With this approximation, the percentage of homoplasmic variants rises to 98%.

**Figure 8:**
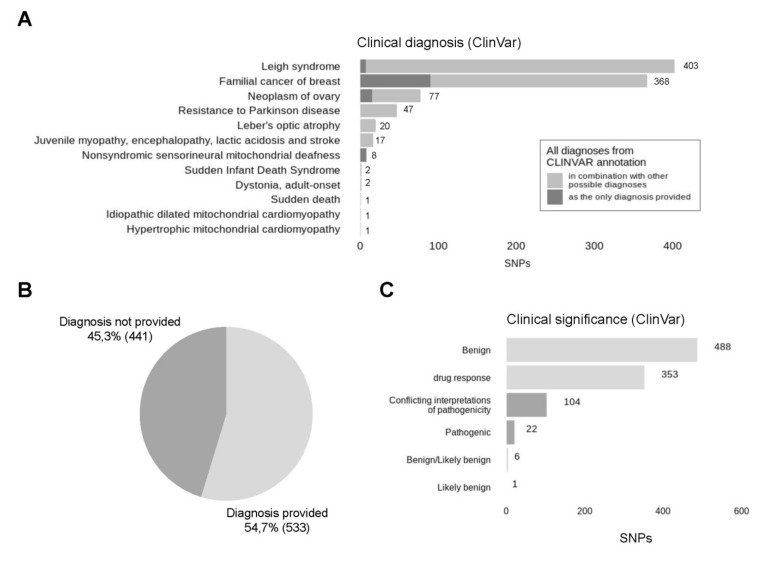
Clinical significance of detected SNPs. (**A**) All clinical diagnoses associated with analysed variants were collected and represented in descending order by the number of occurrences, cases, where the found diagnosis was the only diagnosis provided for this particular variant, are presented in dark grey colour, while the ones where the diagnosis was provided in combination with other possible interpretations are presented in light grey colour. (**B**) In total, clinical diagnosis was provided for 68.7% of analysed variants. (**C**) Distribution of detected SNPs by their clinical significance according to ClinVar. Light grey represents likely benign variants, dark grey represents pathogenic variants.

Analysis of clinical diagnoses associated with discovered variants has revealed that most of the variants were annotated with several different, sometimes even conflicting, diagnoses, because ClinVar database is constantly supplemented. Distribution of distinct diagnoses is presented in Figure 8A. As expected, Leigh syndrome (38.8% of all patients) and Familial cancer of breast (38.6% of patients) are the most frequent diagnoses since they are presented by a large number of variants in ClinVar database. Five different variants are associated with familial cancer of breast—rs2853508 (85% of all variants associated with familial cancer of breast), rs193302980 (6.5%), rs527236177 (3.3%), rs193302983 (3.3%), rs193302985 (1.6%). The first three variants are also linked with Leigh syndrome, according to new data. In total, Leigh syndrome was assigned to 12 distinct variants, most of which previously had another interpretation. Next, there are Neoplasm of ovary (8.4%), resistance to Parkinson disease (4.4%), Leber’s optic atrophy (2.6%) and Juvenile myopathy, encephalopathy, lactic acidosis and stroke (1.6%). The remaining six diagnoses are represented with isolated variants and account for less than 1% of all variants. In total, 54.7% of variants analysed are provided with a clinical diagnosis from ClinVar (Figure 8B). Most of the variants without any diagnosis provided (45.3%) are presumably associated with drug response. Drug response is the second-largest category, including 37.3% of all variants, but possible diagnoses associated with these variants are not discovered yet (Figure 8C). Most of the variants (51.5%) are benign, 11% have conflicting interpretations of pathogenicity and only 2.3% (22) are pathogenic according to ACMG criteria. Among 22 pathogenic variants, 8 (36.4%) are associated with nonsyndromic sensorineural mitochondrial deafness (m.961T>G), one is reported to be associated with neoplasm of ovary (m.15511T>C) and 13 (59%) do not have clinical diagnosis provided: m.951G>A (4), m.980T>C (1), m.1008A>G (2), m.8410C>T (1), m.11560A>G (1), m.14470T>A (1), m.15262T>C(1), m.15514T>C (1) and m.15833C>T (1). 

However, considering the high frequency of these variants among our samples, their pathogenicity is questionable.

## 4. Discussion

In this study, we explored the quantitative and qualitative composition of mtDNA pool of maternal cfDNA fraction from pregnant women, investigated how this composition is affected by transportation and storage conditions and experimented with utilising cf-mtDNA to conduct mitochondrial analyses.

The sequencing of buffy coat DNA from selected samples has revealed that mtDNA pools from cfDNA and buffy coat do barely intersect. However, our result supports previous findings on the variability of mtDNA variant numbers between different tissues [10]. Since the risk of NUMT contamination is negligible, the observed differences may arise from the origin of DNA fragments in these pools since mtDNA of buffy coat is mostly presented by the mtDNA fragments of intact blood cells while the composition of cf-mtDNA fraction is more complex and includes foetal mtDNA and heterogeneous maternal mtDNA of apoptotic cells from different organs and systems of the body. In particular, the predominantly apoptotic origin of mtDNA fragments in cf-mtDNA fraction can be a possible explanation for the different set of variants in cf-mtDNA fraction, because apoptosis is known to be a common cell response to incorrect DNA replication or DNA damage [65]. The proximity of mtDNA to possible oxidative stress can cause accumulation of repair mismatches in mtDNA sequence [66,67] and lead to cell death that allows alternate variants of mtDNA to add to cfDNA fraction in plasma. Moreover, some technical artefacts could not be discounted. 

According to our data, the differences in the variant sets between cf-mtDNA and buffy coat DNA are observed in both Streck and EDTA samples. Generally, mtDNA content in Streck samples is higher due to degradation of leukocytes, so we observe more shared mitochondrial variants between cfDNA and buffy coat. In EDTA samples, conversely, we detect a smaller set of mtDNA variants. This can be attributed to low sequencing depth and incomplete mtDNA coverage resulting from low mtDNA content in EDTA samples. Our results allow us to conclude that, technically, Streck samples provide a more complete picture of mtDNA.

We showed that the size and composition of the mtDNA pool in samples are heavily dependent on conditions and time of transportation. Streck sample tubes are widely accepted and validated as a standard for NIPT [68]. Moreover, results by Hidestrand et al. present that blood collected into Streck BCT tubes had the total DNA level not changed within 72 h if shipped without freezing [63]. Still, the real shipping speed from distant regions such as Yakutia might be much slower and we show that mtDNA content is significantly increasing over time above the 72 h threshold (Figure 4C). Our results suggest that degrading blood cells might be the main contributors to the mtDNA pool in samples subjected to long transportation. Maternal contamination is a major challenge in prenatal diagnosis [69,70], however, the tracking of contamination is not yet a common practice. Here we speculate that measuring the mtDNA level might allow us to make a rough estimate of the extent of sample contamination with degraded blood cells and check the purity of plasma which is important for foetal fraction estimation as an essential NIPT quality control component [71]. The low foetal fraction may be responsible for up to 50% of all failures as the excess of normal maternal DNA with a double set of chromosomes can mask the foetal aneuploidies and lead to false-negative results [72,73]. Here we show that shipping over 3 days results in higher mtDNA rate in samples, and the increase in mtDNA content leads to lower foetal fraction level thus potentially impacting NIPT results quality. There are techniques allowing the detection of maternal contamination in NIPT, such as single nucleotide polymorphism (SNP) oligonucleotide microarray analysis (SOMA) [74]. However, this method requires additional sequencing of a maternal sample which leads to increase in costs. Our results suggest mtDNA as a contamination marker that can be used for quality control to evaluate the extent of maternal cells degradation in samples. 

The contamination effect must also be taken into account when using mtDNA content as a diagnostic sign of inflammation and other pathologies since blood cell mtDNA contamination can result in misleading measurements. However, increased share of blood cell DNA in cfDNA does not preclude population studies but is undesirable during NIPT since it can distort foetal fraction measuring. 

We analysed mtDNA from 645 cfDNA samples and called over 7000 distinct variants, explored a set of mitochondrial variants called from cfDNA and tested its applicability for clinic and research purposes. Within the cell, the mtDNA-gDNA content ratio is less then 1:1000 [75], which is generally in line with mtDNA content we detected in cfDNA fraction (Figure 1A). It must be noted that while traditional genetic and clinical tests based on mtDNA analysis are conducted on amplified or deeply sequenced mtDNA [75,76], conducting similar tests within NIPT places significant limits caused by low coverage of cf-mtDNA [77]. Nevertheless, our results indicate that we can perform some mtDNA tests despite low coverage. The quantitative analysis of mtDNA pool has revealed that mtDNA in all samples with mean mtDNA sequencing depth exceeding 2× (43.7%) has more than 75% coverage, moreover, samples with mean mtDNA sequencing depth exceeding 4× (17.6%) have more than 95% of mtDNA covered (data not shown). To reach full mtDNA coverage, mean sequencing depth of mtDNA should be higher than 7–8. The main advantage of our approach is that it does not require any additional steps of sample preparation and mtDNA amplification—we get results of mtDNA analysis as a by-product of an ordinary WGS NIPT protocol. 

We checked the distribution of variants throughout the mitochondrial genome and revealed some hot and cold spots of variation. As expected, variants are accumulating within the control region of mtDNA supporting previous studies [39], but we also detected hotspots of variation outside of this region. Apart from non-coding regions, variants are accumulating in intergenic regions between 4 and 5, 11 and 13 kb. Regions between 5 and 7, 8 and 10 kb carry significantly fewer variants. In order to figure out the reasons for this heterogeneity, we summed up all samples and explored the distribution of total coverage throughout the mitochondrial genome (data not shown). Cold spot regions have lower coverage, suggesting that some fragments of mtDNA are less frequent in cfDNA, probably due to GC sequencing bias [78] and selective mtDNA fragmentation [79]. 

Called variants can be used for mitochondrial haplogroup analysis. Haplogroup analysis showed haplogroups H (40.5%), C (13.7%), D (11.1%) and U (11.1%) to be the most common among the explored samples, which is typical for a mixture of Slavic and Asian populations presented in our data [43]. We compared our haplogroup frequencies with previous reports on mtDNA haplogroup frequencies of Russian and European populations. Haplogroup H was reported to be the top frequent mtDNA haplogroup in Russia with a population frequency of 37.3% [80], which is highly consistent with our results—40.5%. Our frequencies of haplogroups J (8.9%), M (1.8%), T (5.2%) and W (1.3%) are generally in line with previously reported frequencies [80,81]. Haplogroup U (11.1%) is less frequent than reported (~17%). The presence of C (13.7%) and D (11.1%) haplogroups can be attributed to mongoloid admixture since mtDNA haplogroups H, U, K, J, V, I and W are typical for west Eurasian populations, while A, B, C, D and M are mostly east Eurasian (Asian) mitotypes [80,82]. Indeed, C and D haplogroups in our data are mostly presented by samples from Yakutia. Moreover, we studied the distribution of the ancestral variants, especially common for Asian mitotypes, throughout different populations and found these variants to be the most frequent among Yakutian samples. 

CfDNA has been already used for identifying mitochondrial haplogroups in previous studies [82]. However, it involved the cf-mtDNA amplification step, while we worked with unamplified cf-mtDNA. In the majority of cases, the set of variants was not enough for full haplogroup determination, so we worked with superclades. It has been empirically identified that reliable haplogroup determination requires hundreds of variants [83], which is also confirmed by our results. However, we can use samples with high mtDNA content, particularly the ones that were enriched with mtDNA as result of long transportation, to determine mitochondrial haplogroups to the level of specific subclades.

From a clinical perspective, mitochondrial SNPs called from NIPT samples are incredibly challenging to interpret. This is attributable to heteroplasmy and so-called ‘bottleneck-effect’ when a low amount of mutated mtDNA in the mother germline can result in a high percentage of mutated mitochondria in the foetus during embryonic development. Mitochondrial mutations occurring in the germline go through the transient heteroplasmic state, which resolves itself within a few generations [84]. In other words, it is not possible to establish any threshold for SNPs to exclude the possibility of disease development in further generations. For genomic SNPs, minor allele frequency (MAF) of less than 5% indicates insignificant SNPs, while for mitochondrial SNPs this threshold should be much lower due to heteroplasmy [85]. It turns out that despite low sequencing depth we still can detect significant SNPs. However, mitochondrial variants called from the cfDNA pool of NIPT samples have mixed origin [12] and therefore their locations within a body cannot be established. Moreover, the accumulation of data on the clinical significance of mitochondrial variants is still in process, which often leads to false-positive results. Taken together, the robust prediction of mitochondrial diseases based on the analysis of variants called from NIPT samples is hardly possible, so clinical use of the method is limited. Still, detection of clinically significant variants might be important as a preliminary screening that can be an additional reason to direct a patient to undergo traditional mtDNA tests but cannot be considered as a self-sufficient clinical test for mitochondrial disease.

Nevertheless, in spite of limited diagnostic capability, called variants can still be used in population studies. The analysis of population frequencies of called variants showed that ancestral variants [62] m.15326A<G (22.3%), m.750A>G (20.9%), m.2706A>G (18.8%) and m.263A>G (15.0%) are the most common among studied patients. Frequent non-ancestral variants are presented with microdeletions and microinsertions: m.9906delG (12.6%), m.10151delA (6.8%), m.9916delC (5.6%), m.2193delT (4.3%), m.9808insT (4.2%).

NIPT is becoming a routine screening method and more and more samples are being tested annually all over the world. Our study is proof of principle that confirms the possibility of using NIPT samples in studies based on mtDNA analysis. MtDNA can be easily obtained from cfDNA sequencing data during the NIPT test and can be potentially used for haplogroup determination, quality control or stored for further studies. Currently, we do not have enough data to make valid clinical predictions, there must be a significant increase in sample size to develop the methods of mtDNA-based clinical diagnosis. Given the increasing popularity of NIPT, mtDNA from cfDNA data is a promising research object, which can add to our knowledge of the population structure and the possible pathogenicity of variants with unclear clinical significance.

## Figures and Tables

**Table 1:** The number of point mutations (SNPs) and indels before and after two-step filtration.

	Total Number of Variants	Indels	SNPs
Total	32,962	21,416	11,546
Depth filtering	22,017	17,035	4982
Homopolymer filtering (≥4)	14,681	10,479	4202
Homopolymer filtering (≥3)	8969	6450	2519

**Table 2:** Top-5 most frequent non-ancestral point variants. Columns from left to right: mtDNA sequence variant, variant frequency (number of patients and frequency).

mtDNA Variant	Patients
m.15301G>A	43 (6.7%)
m.489T>C	36 (5.6%)
m.10400C>T	36 (5.6%)
m.14783T>C	34 (5.3%)
m.15452C>A	29 (4.5%)

**Table 3:** Top-5 most frequent non-ancestral indels. Columns from left to right: mtDNA sequence variant, number of patients and frequency.

mtDNA Variant	Patients
m.9906delG	81 (12.6%)
m.10151delA	44 (6.8%)
m.9916delC	38 (5.6%)
m.2193delT	28 (4.3%)
m.9808insT	27 (4.2%)

**Table 4:** Top-5 most frequent non-ancestral SNPs presented in ClinVar database. Columns from left to right: mtDNA sequence variant, variant ID (rs), variant ID (ClinVar), clinical significance (ClinVar), diagnosis (ClinVar), number of patients and frequency.

mtDNA Variant	rs ID	ClinVar ID	Clinical Significance	Diagnosis (ClinVar)	Number of Patients
m.15301G>A	193302991	140591	Conflicting interpretations of pathogenicity	Familial cancer of breast	43 (6.7%)
m.14783T>C	193302982	140588	Conflicting interpretations of pathogenicity	Familial cancer of breast	34 (5.3%)
m.15452C>A	193302994	143925	Benign	Neoplasm of ovary/ Leigh syndrome	29 (4.5%)
m.3010G>A	3928306	441149	Drug response	Not provided	28 (4.3%)
m.13708G>A	28359178	9696	Benign	Leber’s optic atrophy/Leigh syndrome	10 (1.6%)

**Table 5:** Top-3 non-ancestral variants that are frequent in Yakutia and rare in other regions. Columns from left to right: mtDNA sequence variant, the frequency of the variant in each region (% from the number of samples in each region), the total number of patients carrying the variant.

DNA Seq Variant	Central (%)	Northwestern (%)	Volga-Ural (%)	Yakutia (%)	Others (%)	Number of Patients
m.15301G>A	1.81	8.00	0.0	79.17	0.00	54 (8.4%)
m.10400C>T	0.00	2.67	2.5	75.00	0.00	41 (6.4%)
m.12704TC>T	1.36	3.33	0.0	47.92	0.00	31 (4.8%)

## Data Availability

The data are not publicly available due to due to commercial restrictions.

## Supplementary Materials

The following are available online at https://www.mdpi.com/article/10.3390/genes12050743/s1, Figure S1: Distribution of variants throughout the mitochondrial genome before (a) and after (b) filtering of homopolymers.
