# Supplementary material for: Pilot Screening of Cell-Free mtDNA in NIPT: Quality Control, Variant Calling, and Haplogroup Determination
Source: Genes (Basel). 2021 May 14;12(5):743. doi: 10.3390/genes12050743 (PMC8156457; doi:10.3390/genes12050743)
Supplement: Supplementary file 1 [file genes-12-00743-s001.zip › genes-1137844-supplementary.pdf]

genes

MDPI

Article

Pilot Screening of Cell-Free mtDNA in NIPT: Quality Control, Variant Calling and Haplogroup Determination

Alisa Morshneva[1,2*], Polina Kozyulina[1,2], Elena Vashukova[1,2], Olga Tarasenko[1,2], Natalia Dvoynova[2], Anastasia Chentsova[2], Olga Talantova[1], Alexander Koroteev[3,4], Dmitrii Ivanov[3], Elena Serebryakova[1], Tatyana Ivashchenko[1], Aitalina Sukhomyasova[5,6], Nadezhda Maksimova[6], Olesya Bespalova[1], Igor Kogan[1], Vladislav Baranov[1] and Andrey Glotov[1,2]

Supplementary Materials

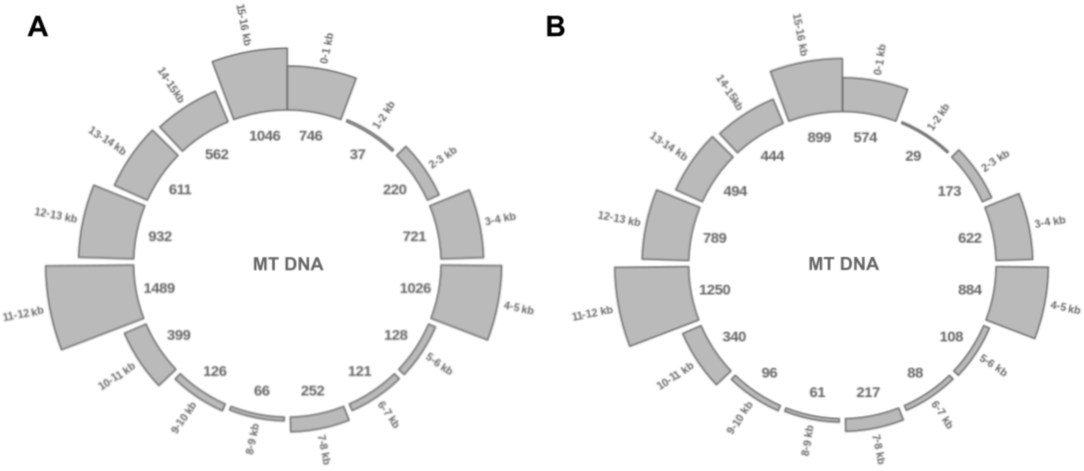

Figure S1. Distribution of variants throughout the mitochondrial genome before (A) and after (B) filtering of homopolymers. Figures outside the circle mark position in the mitochondrial genome (16kb mtDNA has been divided into 16 sections per 1kb), figures in the inner circle represent the number of variants in every section.
